# Using the ratio of means as the effect size measure in combining results of microarray experiments

**DOI:** 10.1186/1752-0509-3-106

**Published:** 2009-11-05

**Authors:** Pingzhao Hu, Celia MT Greenwood, Joseph Beyene

**Affiliations:** 1 The Centre for Applied Genomics, The Hospital for Sick Children, 15-706 TMDT, 101 College Street, Toronto, ON, M5G 1L7, Canada; 2 Dalla Lana School of Public Health, University of Toronto, Health Sciences Building, 155 College St, Toronto, ON, M5T 3M7, Canada; 3 Child Health Evaluative Sciences, The Hospital for Sick Children Research Institute, 555 University Ave, Toronto, ON, M5G 1X8, Canada

## Abstract

**Background:**

Development of efficient analytic methodologies for combining microarray results is a major challenge in gene expression analysis. The widely used effect size models are thought to provide an efficient modeling framework for this purpose, where the measures of association for each study and each gene are combined, weighted by the standard errors. A significant disadvantage of this strategy is that the quality of different data sets may be highly variable, but this information is usually neglected during the integration. Moreover, it is widely known that the estimated standard deviations are probably unstable in the commonly used effect size measures (such as standardized mean difference) when sample sizes in each group are small.

**Results:**

We propose a re-parameterization of the traditional mean difference based effect measure by using the log ratio of means as an effect size measure for each gene in each study. The estimated effect sizes for all studies were then combined under two modeling frameworks: the quality-unweighted random effects models and the quality-weighted random effects models. We defined the quality measure as a function of the detection p-value, which indicates whether a transcript is reliably detected or not on the Affymetrix gene chip. The new effect size measure is evaluated and compared under the quality-weighted and quality-unweighted data integration frameworks using simulated data sets, and also in several data sets of prostate cancer patients and controls. We focus on identifying differentially expressed biomarkers for prediction of cancer outcomes.

**Conclusion:**

Our results show that the proposed effect size measure (log ratio of means) has better power to identify differentially expressed genes, and that the detected genes have better performance in predicting cancer outcomes than the commonly used effect size measure, the standardized mean difference (SMD), under both quality-weighted and quality-unweighted data integration frameworks. The new effect size measure and the quality-weighted microarray data integration framework provide efficient ways to combine microarray results.

## Background

Microarray technology has been widely used in identifying differentially expressed genes [1,2] and in building predictors for disease outcome diagnosis [3-7]. Although individual microarray studies can be highly informative for this purpose (e.g. van 'tVeer et al., [4]), it is difficult to make a direct comparison among the results obtained by different groups addressing similar biological problems, since laboratory protocols, microarray platforms and analysis techniques used in each study may not be identical [8,9]. Moreover, most individual studies have relatively small sample sizes, and hence prediction models trained on individual studies by using cross-validation procedures are prone to over-fitting, leading to prediction accuracies that are over-estimated and lack generalizability [10].

Recent studies show that systematic integration of gene expression data from different sources can increase statistical power to detect differentially expressed genes while allowing for an assessment of heterogeneity [11-18], and may lead to more robust, reproducible and accurate predictions [19]. Therefore, our ability to develop powerful statistical methods for efficiently integrating related genomic experiments is critical to the success of the massive investment made on genomic studies. Broadly speaking, the strategies to integrate microarray studies can be divided into three categories:

The first category is a combined analysis of all the data. Each data set is first preprocessed to clean and align the signals, and then these preprocessed datasets are put together so that the integrated data set can be treated as though it comes from a single study. In this way, the effective sample size is greatly increased. Several transformation methods have been proposed to process gene expression measures from different studies [9,14,17,20]. For example, Jiang et al. [14] transformed the normalized data sets to have similar distributions and then put the data sets together. Wang et al. [17] standardized gene expression levels based on the means and standard deviations of expression measurements from the arrays of healthy prostate samples. These methods are simple and in many cases, if the transformation is carefully made, the performance of disease outcome prediction can be improved [14]. Nevertheless, there are no consensus or clear guidelines on the best way to perform the necessary data transformations.

The second strategy is to combine analysis results obtained from each study. The basic idea is to combine evidence of differential expression using a summary statistic, such as the p-value, across multiple gene profiling studies and then to adjust for multiple testing. For example, Rhodes et al. [11,12] combined results from four prostate cancer microarray datasets analyzed on different platforms. Differential expression between the prostate tumor group and the normal group was first assessed independently for each gene in each dataset using the statistical confidence measure, the p-value. Then the study-specific p-values were combined, using the result that -2 log(p-value) has a chi-squared distribution under the null hypothesis of no differential expression. The analysis revealed that stronger significance was obtained from the combined analysis than from the individual studies. Combining p-values is useful in obtaining more precise estimates of significance, but this method does not indicate the direction of significance (e.g., up-or down-regulation) [21]. Instead of integrating p-values directly, some studies explored combining ranks of statistics from different studies [18,22]. For example, DeConde et al. [22] proposed a rank-aggregation method to combine final microarray results from five prostate cancer studies. The method summarizes majority preferences between pairs of genes across ranked list from different studies. They found this method more reliably identifies differentially expressed genes across studies.

The third strategy involves taking inter-study variability into account when estimating the overall effect for each gene across studies, and then basing conclusions on the distribution of these overall measures. For example, Choi et al. [13] focused on integrating effect size estimates in individual studies into an overall estimate of the average effect size. The effect size is normally used to measure the magnitude of treatment effect in a given study. Inter-study variability was included in the model with an associated prior distribution. This type of model, also termed hierarchical Bayesian random effects, has been used broadly in non-microarray contexts (e.g., DuMouchel and Harris [23]; Smith et al., [24]). Using the same microarray datasets as those used by Rhodes et al. [11], they demonstrated that their method can lead to the discovery of small but consistent expression changes with increased sensitivity and reliability among the datasets. The hierarchical Bayesian random effects meta-analysis model has several favorable features: it provides an overall effect size, and it accounts for inter-study variability, which may improve accuracy of results.

The widely used effect size measure in this type of models is the standardized mean difference [25,26]. It has been well-known in microarray data analysis that the estimated standard deviation is probably unstable when sample size in each group is small. Therefore, many efforts have been made to overcome the shortcoming by estimating a penalty parameter for smoothing the estimates using information from all genes rather than relying solely on the estimates from an individual gene [1,27].

However, recent studies show that differentially expressed genes may be best identified using fold-change measures rather than t-like statistics [28]. Fold change is a commonly used measure in small laboratory experiments of gene expression; it is considered to be a natural measure for gene expression changes [29]. In high-throughput microarray analysis, properties of fold change statistics have received little attention. Therefore, more investigation on reparameterization of effect size measures is needed.

Most data integration papers in microarray analysis have not used measures of quality to refine their analyses [9,11-15,17,20,22]. Nevertheless, in classical meta-analysis, quality measures have often been used when combining results across studies. It has been argued that studies of a higher quality will give more accurate estimates of the true parameter of interest, and therefore studies of high quality should receive a higher weight in the analysis summarizing across studies [30]. In gene expression microarrays, many genes may be "off" or not detectable in a particular adult tissue, and in addition, some genes may be poorly measured due to probes that are not sufficiently sensitive or specific. Therefore, the signal strength and clarity will vary across the genes, suggesting that a quality measurement could highlight strong clear signals [31,32]. Although it is still an open question how to best measure the quality of a gene expression measurement, and how best to use such a quality measure, different strategies can be considered for incorporating quality weights into meta-analysis of microarray studies. For example, we can define a quality threshold and only include genes that are above this threshold in the meta-analysis. However, the choice of threshold will be arbitrary. In a recent study, we proposed a quality measure based on the detection p-values estimated from Affymetrix microarray raw data [16,31]. Using an effect-size model, we demonstrated that the incorporation of quality weights into the study-specific test statistics, within a meta-analysis of two Affymetrix microarray studies, produced more biological meaningful results than the unweighted analysis [16].

In this paper, we reparameterize the effect size measure for each gene in each study as the log ratio of the mean expressions of the two groups being compared. Following the method proposed by Hu et al. [16], we then place the new effect size measure into a quality-weighted modeling framework. We evaluate and compare the effect size measures (new and old) under the quality-weighted and quality-unweighted data integration frameworks using simulated data sets and real data sets with focus on identifying differentially expressed biomarkers and their performance on cancer outcome prediction.

## Methods

### Quality score measure for Affymetrix microarray data

For Affymetrix expression data, we previously developed a quality measure based on the detection p-values [33] that reflects whether the transcript is reliably expressed above the background in at least one experimental group in each study [16,31] (see Additional file 1). The sensitivity parameter, *v*, that alters the tolerance of the quality weight to the detection p-value significance levels, was set to 0.05.

### Using log ratio of means as effect size measure

There are many ways to measure effect size for gene *g *in individual study [25]. A commonly used way is the standardized mean difference (SMD). Let *r*_*gl *_represent the raw expression value for gene *g *and subject *l *and *x*_*gl *_= log(*r*_*gl*_). The standardized mean difference (SMD) of *x*_*gl *_is given by(1)

where  and  are the sample means of logged gene expression values for gene *g *in treatment group (t) and control group (c) in a given study, respectively.  is the pooled standard deviation for gene g. The estimated variance  of the unbiased effect size *y*_*g*1 _is given by Cooper and Hedges [25](2)

For a study with *n*(*n *= *n*_*t*_+*n*_*c*_) samples, an approximately unbiased estimate of *y*_*g*1 _is given by [26].

Here, we propose an alternative method to measure effect size based on the log ratio of means (ROM), that is, the log fold-change given by(3)

In contrast to the previous approach, here  and  are the sample means of unlog -transformed gene expression values for gene *g *in treatment group (t) and control group (c) in a given study, respectively. The estimated variance  of the effect size *y*_*g*2 _can be estimated using delta method [34] as follows(4)

where  and  are the variances of the treatment and control groups, respectively.

### Integrative analysis of effect sizes in a quality-adjusted modeling framework

Any defined quality measure can be incorporated into integrative analysis of gene expression profiles using a quality-adjusted meta-analysis framework [16]. The rationale of the framework is that studies of a high quality should receive a higher weight in the analysis summarizing across studies [30]. Here, we follow Hu et al. [16] to place either the SMD effect size measure *y*_*g*1_or the ROM effect size measure *y*_*g*2 _into a hierarchical model and to test for differences between groups. For either measure, we can write, for study *i *and measure *m *(*m *∈ SMD or ROM),(5)

where  is the between-study variability of gene *g *with effect size measure *m*, *μ*_*gm *_represents the average measure of differential expression across the *I *studies for gene *g*. Here,  and *μ*_*gm *_are gene-specific while  and *y*_*igm *_are gene and study-specific (*i = 1,2,...,I*). The quantity  measures the effect size variance of gene g, measuring the sampling error for the *i*^*th *^study. Following Hu et al. [16], we can estimate *μ*_*gm *_by taking the quality *q*_*ig *_for gene *g *and study *i *into account(6)

where *q*_*ig *_and *y*_*igm *_are quality measure and the estimated effect size based on measure *m *for gene *g *in study *i*, respectively.  and  is the between-study variability [13]. Here we used a random-effects model to combine the estimated effect sizes (see Additional file 1). The variance of this estimator is obtained by(7)

A test statistic to evaluate differential expression of gene *g *across all *I *studies can then be computed as(8)

We evaluated the statistical significance of gene *g *by calculating the p-value corresponding to the z statistic; then we estimated the false discovery rates (FDR) for each significance level, to take into account the number of tests performed [35]. A detailed description of the integrative analysis of effect sizes can be found in the see Additional file 1.

We refer the approaches of estimating *z*_*gm *_using either the log ratio of means (*m *= 2) or the standardized mean difference (*m *= 1) as WROM and WSMD, respectively, in the quality-adjusted modeling framework, and as UWROM and UWSMD, respectively, in the quality-unadjusted modeling framework, where *q*_*ig *_= 1.

### Simulations

#### Model probe-level gene expression profile in a single study

Following previous studies to generate Affymetrix probe level data [31,36], we modeled the probe-level gene expression for different conditions (e.g. cancer and normal samples) in a single study as:(9)

where *Y*_*jgk *_and *W*_*jgk *_are *PM *and *MM *intensities for the probe *j *in probeset *g *on array *k *respectively. *O *denotes optical noise, independently drawing from  and [36].  represent non-specific binding (NSB) noise for *PM *(*XX = PM*) and *MM *(XX = *MM*), respectively. We set *μ*^*MM *^= *μ*^*PM *^= 4.6 and assumed that  and  follow a bivariate normal distribution with mean 0, variance 1, and correlation 0.88. We then generated identically and independently distributed random variates *e *~ *N*(0,0.08), so that  and similarly .  are quantity proportional to RNA expression for *PM *(*XX = PM*) and *MM *(XX = *MM*), respectively, and the coefficient 0 < Φ < 1 accounts for the fact that for some probe-pairs the *MM *detects signal; When probe *j *of gene *g *is attached by picking up stray signal, Φ_*jg *_is generated as Φ_*jg*_~*Beta*(0.5,5), otherwise, Φ_*gj *_= 0. Since S follows a power law, we set its base to 2. Therefore, if we denote *γ*_*g *_as the baseline log expression level for probeset *g*, we can select log_2_(*γ*_*g*_) expression levels from 0 to 12, which can be generated from *γ*_*g*_~12* *Beta*(1,3)+1. *δ*_*g *_is the expected differential expression of gene *g *in covariate *X*. *α*_*jgk *_is the signal detecting ability of probe *j *in gene *g *on array *k*, which is assumed to follow a normal distribution with mean zero and signal detection variance . We generated multiplicative errors  and  independently from N(0, ).

#### Generate simulated data sets for multiple studies

We generated two Affymetrix microarray data sets, which are assumed to be from two independent studies. In each of the two data sets, we assume treatment group *t *and control group *c *with  and  arrays in the *i*^*th *^study, respectively. We generated *G *genes and assume the proportion of expressed genes is *q *and the proportion of differentially expressed genes is *d *of the G*q expressed genes in each study. We ran three simulation models following the above design by varying treatment effects on the signal between 1.0 (small) and 2 (large) with interval 0.5. The specific parameters used in the five models are summarized in Table 1:

**Table 1:** Parameters used in simulation of probe-level gene expression profile

Parameter	Study 1	Study 2
Number of genes	1000	1000

Proportion of expressed genes	0.5	0.5

Proportion of differentially expressed genes	0.1	0.1

Sample size	25 arrays in groups *t *and *c*, respectively	50 arrays in groups *t *and *c*, respectively

Number of probes in each probeset	11	16

We used summarized receiver operating characteristic (SROC) curves to compare performance, where the test sensitivities and specificities (true positive and true negative proportions) for a range of p-value cutoffs were averaged over 500 simulated datasets in each study. The SROC curve's overall behavior can be measured by the area under the curve (AUC) [37].

### Affymetrix Microarray data

We used gene expression data on prostate tumours and controls from four studies [38-41]. The datasets will be referred to by the name of the first author. All these datasets are either publicly available or obtainable upon request. Information about these datasets, such as microarray platforms, the number of samples available, etc, is listed in Table 2. For these four data sets, we used the robust multi-array average (RMA) algorithm [42] to get summarized probeset-level expression data, and then we obtained the unlogged normalized expression data. There are 12,600 common probesets across the four data sets. We performed integrative analysis using the first three data sets in the table (the Welsh data, the LaTulippe data, and the Singh data) to identify differentially expressed genes and then developed our predictive models (the "training data") based on the selected genes. The fourth data set (the Stuart data) was used for testing the models (the "testing" data).

**Table 2:** Main characteristics of the Affymetrix microarray data sets

Studies	Number of Normal Samples	Number of Prostate Cancer Samples	Chip Type
Singh study (Singh et al., 2002)	50	52	Affymetrix (HG_U95Av2)

Welsh study (Welsh et al., 2001)	8	25	Affymetrix (HG_U95Av2)

LaTulippe study (LaTulippe et al., 2002)	3	23	Affymetrix (HG_U95Av2)

Stuart study (Stuart et al., 2004)	50	38	Affymetrix (HG_U95Av2)

## Results

### Analysis of simulated data sets

We evaluated the performance of our method using simulated Affymetrix probe level expression data generated from a model incorporating probe level effects, optical noise, and non-specific binding, as well as true signals [31,36]. Following the simulation procedures described in Methods section, we run three simulation models for probe-level gene expression profiles generated from two independent studies. Treatment effects on the signal were varied between 1.0 (small) and 2.0 (large) in the three models. Table 3 shows AUCs for the three simulation models under different weighting and effect size parameterization strategies. As seen from the table, the quality-weighted data integration framework produces better performance than the quality-unweighted data integration framework for SMD and ROM-based effect size (It should be noted that the normalized gene expression values for SMD and ROM-based effect sizes are given in log2 and natural scale, respectively), respectively. In terms of the effect size measures, the proposed log ratio of mean method has higher sensitivity than the standardized mean difference method.

**Table 3:** Area under the curves of the four meta-analysis models (*s *= 0.05)

Effect Size	WROM	UWROM	WSMD	UWSMD
*δ*_*g *_= 2.0	0.978	0.965	0.942	0.903

*δ*_*g *_= 1.5	0.962	0.949	0.942	0.905

*δ*_*g *_= 1.0	0.958	0.932	0.924	0.877

### Analysis of prostate cancer Affymetrix microarray data sets

#### Comparing gene ranks among different meta-analytic procedures

To evaluate the significance of genes identified by quality-adjusted and quality-unadjusted data integration frameworks under ROM and SDM effect size measures, we compared the ranks of a set of known prostate tumor genes. This set of prostate cancer genes are from two sources: The first one is from Welsh study [38], where they discussed four prostate tumor markers or experimentally validated genes in detail (see page 5977 of their paper); the second one is from Tricoli study [43]. In this study, they surveyed the potential markers in prostate cancers diagnosis and presented a detailed analysis of five of them, which were believed to be the most likely candidates. Here we compared the ranks of the nine genes selected by each of the four meta-analysis methods as shown in Table 4. Comparing WROM with WSMD, seven of the nine genes selected by WROM have better ranks (ranked on the top) than those selected by WSMD. Comparing UWROM with UWSMD, six of the nine genes selected by UWROM have better ranks than those selected by UWSMD. This suggests the genes selected by ROM-based meta-analytic frameworks (quality-adjusted and quality-unadjusted) might be more biologically interesting than those selected by SMD-based meta-analytic frameworks.

**Table 4:** Rank of known and validated prostate cancer markers

Gene Name	LIMMA	WROM	UWROM	WSMD	UWSMD	Source
HEPSIN	2	2	2	1	6	Welsh et al. (2001)

MIC-1 (GDF15)	145	19	66	110	61	Welsh et al. (2001)

FASN	173	15	13	72	231	Welsh et al. (2001)

TACSTD1	32	6	6	289	413	Welsh et al. (2001)

PSCA	10344	8948	8072	11622	12259	Tricoli et al. 2004

PSMA	509	508	294	433	220	Tricoli et al. 2004

TERT	6636	4625	7741	9945	7596	Tricoli et al. 2004

GSTP1	1744	99	366	1508	2368	Tricoli et al. 2004

GRN	6386	1880	840	2332	2336	Tricoli et al. 2004

It should be noted that some of the known tumor genes identified by our new methods have much better ranks than the conventional methods. For example, the ranks of tumor genes FASN and TACSTD1 are 15 and 6 by WROM and 13 and 6 by UWROM while the ranks of these genes are 72 and 289 by WSMD and 231 and 413 by UWSMD.

**Figure 1:**
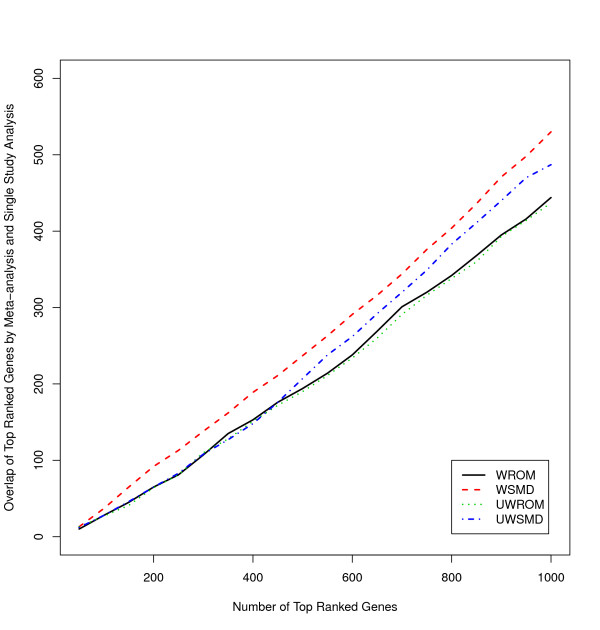
**Comparison of meta-analysis and single study analysis**. Overlap of top-ranked genes by meta-analysis and single study analysis

In order to evaluate the overlap between genes identified by our meta-analysis procedures and those identified in a single study, we analyzed each of the three training data sets (Singh study, Welsh study and LaTulipper study) as shown in Table 2 using LIMMA (LIMMA: linear models for microarray data analysis), a widely used method for identifying differentially expressed genes in a single study [2]. Here we report results using data from Singh's study because this study has relatively large sample size (50 normal and 52 tumor samples). Table 4 and Figure 1 show comparison of results identified from analyzing Singh study alone and those from a meta-analysis of the three studies. As shown in Table 4, the ranks of the 9 known tumor genes based on only Singh study are relatively low and closer to those based on SMD-based meta-analysis procedures than those based on the ROM-based meta-analysis procedures, suggesting ROM-based meta-analysis procedures may have better performance than SMD-based meta-analysis procedures. Therefore, it is not surprising that the overlap between genes identified by LIMMA and our SMD-based meta-analysis procedures is higher than those identified by ROM- based meta-analysis procedures as shown in Figure 1.

#### Comparing prediction performance of top-ranked meta-signatures among meta-analytic procedures

**Figure 2:**
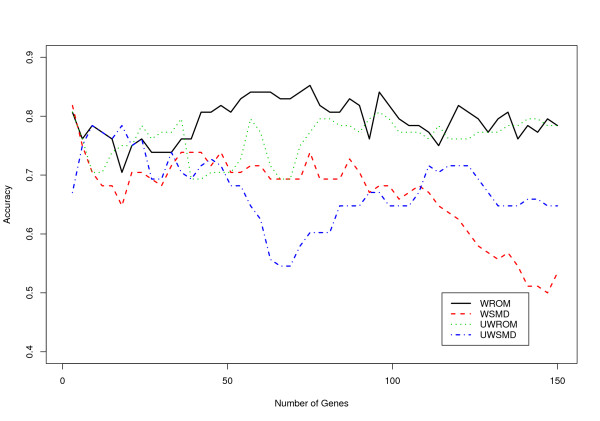
**Prediction accuracy of the SVM model with linear kernel**. Prediction accuracy of the SVM models as a function of the number of differentially expressed genes selected by the four meta-analytic procedures, respectively

**Figure 3:**
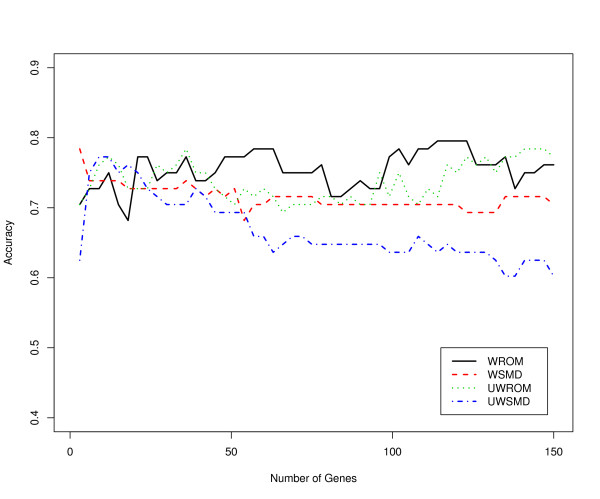
**Prediction accuracy of the SVM model with radial kernel**. Prediction accuracy of the SVM models as a function of the number of differentially expressed genes selected by the four meta-analytic procedures, respectively

To further confirm the validity and biological relevance of the meta-signatures identified by the proposed effect size measures and different data integration frameworks, we evaluated the discriminative power for the top 150 differentially expressed genes identified by the four meta-analysis methods, respectively, using an independent data set listed in Table 2 (Stuart study). We varied the number of predictors between 1 and all the 150 selected genes and built the SVM prediction models on the training dataset listed in Table 2 (Singh study, Welsh study and LaTulippe study), the models were then tested separately for each number of genes included as predictors on the test data (Stuart et al. 2004). Figures 2 and 3 show the classification accuracies based on SVM models with linear and radial kernels, respectively. It can be seen that meta-signatures identified by ROM-based meta-analytic procedures (e.g. WROM and UWROM) usually have better prediction accuracies than those identified by SMD-based meta-analytic procedures (e.g. WSMD and UWSMD). We also tried other simpler classification methods, such as diagonal linear discriminant analysis (DLDA) [5], to build the prediction models, and similar results were observed (data not shown).

## Discussion

Many microarray experiments include only a few replications, therefore, it is critical to improve the effect size estimation in meta-analytic procedure. With small sample sizes, the traditional SMD estimates are prone to unpredictable changes, since gene-specific variability can easily be underestimated resulting in large statistics values. In this study, we re-parameterized the traditional SMD-based effect size measure by using a log ratio of means as an effect size measure for each gene in each study. Our results show the new effect size measure has better performance than the traditional one.

Traditional wisdom for statistical analysis recommends that highly skewed data should be transformed prior to analysis. It is therefore unexpected, perhaps, that the ROM measure (where log transforms are taken after calculating means) gives better prediction accuracy than the SMD measure (where log transformation is done prior to calculating means). Since the signals from Affymetrix are expected to be a mixture of background or non-specific binding and true signal, and only the true signal is expected to follow a power law, using the log transformation up front may be introducing variability, in particular for genes with low levels of expression. Furthermore, for genes whose expression levels change dramatically between experimental groups, the apriori log transformation may be inappropriate in the group with low expression levels.

We noticed that the ranks of some of the known tumor genes (e.g. five candidate markers discussed by Tricoli et al. [43] are relatively low in all four data integration methods (WROM, WSMD, ROM and SMD). There are several possible reasons for this. For example, since the patients used in these studies were collected in different places, there may be clinical heterogeneity, which may result in very different expression profiles of the same gene in different studies. It is also possible that the lower ranks of these tumor genes result from the relatively small sample sizes. Integration of more microarray data sets may lead to the discovery of more robust prostate cancer biomarkers.

Our results show that different predictors, including various combinations of differentially expressed genes can lead to similar prediction accuracy. This can make it challenging to select optimal biomarker sets for clinical use. Our recent study [19] showed that many of the differentially expressed genes which have similar classification results are involved in the same or similar biological pathways. In other words, the genes with the best discriminative power likely correspond to a limited set of biological functions or pathways. Hence, the selection of biomarkers for prediction may need to be based on a combination of statistical results and knowledge of pathways.

It is widely known that data from various sources might contain different informativity for a given biological task (such as differential analysis of gene expression levels between case and control). Some data sources might, for example, be more informative than others. A statistically sound data integration framework should, therefore, take these into account. One approach towards this goal is to develop suitable quality measures for different data types and these measures are then integrated into the statistical models. We used a simple quality measure associated with both log-ratio of means based and standardized mean difference based effect sizes. Our analysis showed this measure works well in the real and simulated data sets.

## Conclusion

In summary, we combined estimated ROM-based effect sizes for all studies under two data integration frameworks: the quality-unweighted random effects models and the quality-weighted random effects models [16]. Comparing with the SMD-based effect size measure, our real examples and simulation studies showed that the proposed methods have better power to identify differential expressed genes and the detected genes have better accuracies in predicting cancer outcomes. In conclusion, the new effect size measure and the quality-weighted microarray data integration framework provide efficient way to combine microarray results.

## List of abbreviations

ROM: ratio of mean; WROM: log ratio of mean used as the effect size measure in weighted meta-analysis Framework; UWROM: log ratio of mean used as the effect size measure in unweighted meta-analysis framework; SMD: standardized mean difference; WSMD: standardized mean difference used as the effect size measure in weighted meta-analysis framework; UWSMD: standardized mean difference used as the effect size measure in unweighted meta-analysis framework; PM: perfect match; MM: mismatch; MLE: maximum likelihood estimation; NSB: non-specific binding; RMA: robust multi-array average; SROC: summarized receiver operating characteristic; AUC: area under the curve; FDR: false discovery rate; SVM: support vector machines; DLDA: diagonal linear discriminant analysis.

## Authors' contributions

JB initiated the study and proposed the ratio of means effect size measure and weighted data integration framework. CG proposed the quality weight measure and simulation framework. PH carried out all the data analysis and drafted the manuscript. All authors read, contributed to, and approved the final manuscript.

## Supplementary Material

Additional file 1 **Data integration methods**. The document describes the data integration methods in detail. Click here for file
