# Supplementary material for: Using the ratio of means as the effect size measure in combining results of microarray experiments
Source: BMC Syst Biol. 2009 Nov 5;3:106. doi: 10.1186/1752-0509-3-106 (PMC2784452; doi:10.1186/1752-0509-3-106)
Supplement: Additional file 1 — Data integration methods. The document describes the data integration methods in detail. [file 1752-0509-3-106-S1.doc]

Using the ratio of means as the effect size measure in combining results of microarray experiments

Pingzhao Hu^1^, Celia MT Greenwood^1,2^ and Joseph Beyene^1,2,3^

^1^ The Centre for Applied Genomics, The Hospital for Sick Children, 15-706 TMDT, 101 College Street, Toronto, ON, M5G 1L7, Canada

^2^ Dalla Lana School of Public Health, University of Toronto, Health Sciences Building

155 College St, Toronto, ON, M5T 3M7, Canada

^3^ Child Health Evaluative Sciences, The Hospital for Sick Children Research Institute, 555 University Ave, Toronto, ON, M5G 1X8, Canada

**Quality score measure for Affymetrix microarray data**

We developed a quality measure based on the detection algorithm proposed by Affymetrix [1] to define the quality of the measurement of a particular transcript in a study [2, 3]. The detection algorithm compares the perfect match (PM) and mismatch (MM) intensities to evaluate whether PM is primarily greater than MM, and whether both numbers are greater than a detection limit, and this algorithm generates a detection p-value. A low detection p-value is an indicator that the perfect match expression signals are consistently larger than the mismatch signals, and hence that the mRNA level is consistently measured. Using these detection p-values, we defined a measure of quality $q_{g}$that applies to gene *g* in each study (for all arrays in that study), built on the assumption that the detection p-values follow an exponential distribution with one-parameter $\lambda_{e}$within experimental group $e(e=1,2,...,E)$in each study. The parameters$\lambda_{e}$for each gene, study and group *e* can be estimated using maximum likelihood estimation (MLE) which has well-known asymptotic optimality properties [4]. Therefore, we can define the quality measure across the groups, for gene *g* in each study as

$q_{g}=\max_{e\in\{1,2,...,E\}}[\exp(\hat{\lambda}_{e}\log v)]$ , (1)

The sensitivity parameter *v* can be varied to alter the tolerance of the quality weight to the detection p-value significance levels. More detail about the definition can be found elsewhere [2, 3]. Our simulation studies [3] showed that an optimal value of *v* should be set to be 0.05. This choice can be thought of as a p-value of 0.05, leading to the interpretation that a representative transcript from a particular experimental group will be called present when the expected (under the assumed distribution) p-value is below 0.05.

**Integrative analysis of effect sizes in a quality-adjusted modeling framework**

To simplify the description, here we drop the subscript *m* (effect size methods) used in main manuscript. Given an effect size measure, for a given gene *g*, let $\mu_{g}$denote its overall mean effect size in all studies, a measure of the average differential expression for that gene. We then redefine the observed effect size$y_{g}$ for gene *g* in study *i* as a hierarchical model:

$$\left\{\begin{matrix} y_{ig}=\theta_{g}+\varepsilon_{ig}\begin{matrix} , & & \varepsilon_{ig}\sim N(0,s_{ig}^{2}) & \end{matrix} \\ \theta_{g}=\mu_{g}+\delta_{g}\begin{matrix} , & & \delta_{g}\sim N(0,\tau_{g}^{2}), & \end{matrix} \end{matrix}\right.$$

where $\tau_{g}^{2}$is the between-study variability of gene *g*. Here, $\tau_{g}^{2}$ and $\mu_{g}$are gene-specific while $s_{ig}^{2}$ and $y_{ig}$are gene and study-specific.

There are two ways to combine the effect sizes from individual studies: fixed effects and random effects models. In essence, in the fixed effects model, the effect sizes in the population are fixed but unknown constants. As such, the effect size in the population is assumed to be the same for all studies included in a meta-analysis. The alternative possibility is that the population effect sizes vary randomly from study to study. In this case each study in a meta-analysis comes from a population that is likely to have a different effect size to any other study in the meta-analysis.

In statistical terms the main difference between these two models is in the calculation of standard errors associated with the combined effect size. In a fixed-effects model (FEM), the within-study variability $s_{ig}^{2}$ in their error term on the observed effect sizes is fully assigned to sampling error only, ignoring the between study variance, so $\tau_{g}^{2}=0$and$y_{ig}\sim N(\mu_{g},s_{ig}^{2})$. On the other hand, a random-effects model (REM) considers that each study estimates a different treatment effect$\theta_{g}$. These parameters are drawn from a normal distribution$\theta_{g}\sim N(\mu_{g},\tau_{^{g}}^{2})$.

To assess whether FEM or REM is most appropriate, we tested the hypothesis$\tau_{g}=$0 using the following test statistic, which is a modification of Cochran’s test statistic [5] by incorporating our quality measure $q_{ig}$ for study *i* and gene *g*

$Q_{g}=\sum_{i}^{I}q_{ig}w_{ig}(y_{ig}-\hat{\mu}_{g})^{2}$,

where $w_{ig}=s_{ig}^{-2}$and

$\hat{\mu}_{g}^{F}=\frac{\sum_{i=1}^{I} q_{ig}w_{ig}y_{ig}}{\sum_{i=1}^{I} q_{ig}w_{ig}}$,

$\hat{\mu}_{g}^{F}$is the weighted least squares estimator that ignores between study variation. Under the null hypothesis of $\tau_{g}=$0, this statistic follows a $\chi_{I-1}^{2}$distribution. We follow Choi et al’s method [6] to draw quantile-quantile plots of $Q_{g}$ to assess whether a FEM or REM model is appropriate. If the null hypothesis of $\tau_{g}=$0 is rejected, we estimate $\tau_{g}$ based on the method developed by DerSimonian and Laird [7].

$\tau_{g}^{2}=\max\left\{0,\left.(Q_{g}-(I-1))/(\sum w_{ig}-(\sum w_{ig}^{2}/\sum w_{ig}))\right\}\right.$,

Therefore, we can estimate $\mu_{g}$

$\hat{\mu}_{g}^{R}=\frac{\sum_{i=1}^{I} q_{ig}w_{ig}^{R}y_{ig}}{\sum_{i=1}^{I} q_{ig}w_{ig}^{R}}$,

where $w_{ig}^{R}=(s_{ig}^{2}+\tau_{g}^{2})^{-1}$. Under the REM,

,

The *z* statistic to test for treatment effect under REM is

,

The *z* statistic for FEM is the same as that for REM except that $\tau_{g}^{2}=$0.

We evaluated the statistical significance of gene *g* by calculating the p-value corresponding to the z statistic, then we estimated the false discovery rates (FDR) for each significance level, to take into account the number of tests performed [8].

**References**

1. Affymetrix – Technical Manual

[<http://www.affymetrix.com/support/technical/manual/expression_manual.affx>].

1. Hu P, Celia GMT, Beyene J: **Integrative analysis of multiple gene expression profiles with quality-adjusted effect size models**. *BMC Bioinformatics* 2005, **6**:128.
2. Hu P, Beyene J, Greenwood CMT: **Tests for differential gene expression using weights in oligonucleotide microarray experiments**. *BMC Genomics* 2006*,* **8**:9-20*.*
3. Knight K: *Mathematical statistics.* Chapman & Hall/CRC Press; 2000.
4. Cochran BG: **The combination of estimates from different experiments*.*** *Biometrics* 1954, **10:** 101-129.
5. Choi JK, Yu U, Kim S, Yoo OJ: **Combining multiple microarray studies and modeling inter-study variation**. *Bioinformatics* 2003, **19 (Suppl.)**: i84-i90.
6. DerSimonian R, Laird NM: **Meta-analysis in clinical trials.** *Controlled Clinical Trials* 1986, **7:**177-188.
7. Benjamini Y, Hochberg Y: **Controlling the false discovery rate: a practical and powerful approach to multiple testing**. *Journal of the Royal Statistical Society: Series* *B* 1995, **85**:289-300.
